# Supplementary material for: Time-series transcriptome analysis identified differentially expressed genes in broiler chicken infected with mixed Eimeria species
Source: Front Genet. 2022 Aug 8;13:886781. doi: 10.3389/fgene.2022.886781 (PMC9393255; doi:10.3389/fgene.2022.886781)
Supplement: Supplementary file 2 [file DataSheet1.ZIP › 4dpi_KEGG.Gsea.1625100298584/KEGG_RETINOL_METABOLISM.html]

Details for gene set KEGG\_RETINOL\_METABOLISM[GSEA]

|  || Dataset | TMM\_4dpi\_gct\_format\_4dpi\_gct\_format.Class\_4dpi.cls #PC\_versus\_NC.Class\_4dpi.cls #PC\_versus\_NC\_repos |
| Phenotype | Class\_4dpi.cls#PC\_versus\_NC\_repos |
| Upregulated in class | 0 |
| GeneSet | KEGG\_RETINOL\_METABOLISM |
| Enrichment Score (ES) | -0.49688575 |
| Normalized Enrichment Score (NES) | -1.5442661 |
| Nominal p-value | 0.03263826 |
| FDR q-value | 0.1591028 |
| FWER p-Value | 0.8626 |
Table: GSEA Results Summary

  

Fig 1: Enrichment plot: KEGG\_RETINOL\_METABOLISM      
 Profile of the Running ES Score & Positions of GeneSet Members on the Rank Ordered List

  

| SYMBOL | TITLE | RANK IN GENE LIST | RANK METRIC SCORE | RUNNING ES | CORE ENRICHMENT || 1 | LRAT | na | 74 | 1.771 | 0.1050 | No |
| 2 | BCO1 | na | 406 | 1.057 | 0.1437 | No |
| 3 | DGAT2 | na | 643 | 0.854 | 0.1776 | No |
| 4 | RDH11 | na | 2004 | 0.422 | 0.0907 | No |
| 5 | RETSAT | na | 2535 | 0.343 | 0.0680 | No |
| 6 | PNPLA4 | na | 3086 | 0.270 | 0.0391 | No |
| 7 | ALDH1A2 | na | 3793 | 0.188 | -0.0080 | No |
| 8 | DHRS3 | na | 5722 | 0.015 | -0.1679 | No |
| 9 | DHRS4 | na | 6556 | -0.051 | -0.2342 | No |
| 10 | CYP3A5 | na | 8491 | -0.228 | -0.3812 | No |
| 11 | CYP4A22 | na | 8776 | -0.259 | -0.3886 | No |
| 12 | CYP3A4 | na | 9602 | -0.362 | -0.4347 | No |
| 13 | ADH6 | na | 9642 | -0.367 | -0.4149 | No |
| 14 | UGT2A1 | na | 10045 | -0.421 | -0.4220 | No |
| 15 | RDH16 | na | 10944 | -0.592 | -0.4597 | Yes |
| 16 | RDH10 | na | 11297 | -0.703 | -0.4449 | Yes |
| 17 | RPE65 | na | 11312 | -0.709 | -0.4016 | Yes |
| 18 | RDH5 | na | 11672 | -0.938 | -0.3726 | Yes |
| 19 | ADH4 | na | 11768 | -1.035 | -0.3156 | Yes |
| 20 | ADH1C | na | 11810 | -1.129 | -0.2481 | Yes |
| 21 | CYP2C18 | na | 11900 | -1.289 | -0.1746 | Yes |
| 22 | UGT1A1 | na | 11907 | -1.313 | -0.0927 | Yes |
| 23 | ALDH1A1 | na | 11955 | -1.613 | 0.0046 | Yes |
Table: GSEA details [plain text format]

  

Fig 2: KEGG\_RETINOL\_METABOLISM      
 Blue-Pink O' Gram in the Space of the Analyzed GeneSet

  

Fig 3: KEGG\_RETINOL\_METABOLISM: Random ES distribution      
 Gene set null distribution of ES for **KEGG\_RETINOL\_METABOLISM**
